# The Association between Shift Work and the Metabolic Syndrome in Female Workers

**DOI:** 10.1186/2052-4374-25-33

**Published:** 2013-11-01

**Authors:** Han Hui Ye, Jae Uk Jeong, Man Joong Jeon, Joon Sakong

**Affiliations:** 1 Department of Occupational and Environmental Medicine, Yeungnam University Hospital, 317-1, Daemyungdong, Namgu, Daegu 705-717, Republic of Korea

**Keywords:** Shift work, Female workers, Metabolic syndrome

## Abstract

**Objective:**

This study aimed to determine identify any association between shift work and the metabolic syndrome by comparing the prevalence rates of the metabolic syndrome in shift work groups and daytime work groups for female workers.

**Methods:**

Based on data from health examinations carried out from April to December of 2012, we selected as our subjects 254 female workers from the Daegu area Dyeing Industrial Complex. We diagnosed the metabolic syndrome using the examination results, and information about age, whether or not they did shift work, job type, smoking habits, drinking habits, exercise habits, and past medical history was collected through self-administered questionnaire surveys and face-to-face interviews. The variables found in a univariate analysis to be significant in the occurrence of the metabolic syndrome - age, drinking habits, exercise habits, and shift work - were included in a logistic regression analysis of the risk of the metabolic syndrome for female workers.

**Results:**

The prevalence rates of the metabolic syndrome for the total group of study subjects was 11.8%, for daytime workers was 2.8%, and for shift workers was 15.3%. A logistic regression analysis of the odds of the metabolic syndrome for female workers was conducted that included factors associated with the occurrence of the metabolic syndrome: age, drinking habits, exercise habits, and shift work. The results revealed that the odds ratio of the metabolic syndrome in the shift work group, 6.30 (95% CI 1.24-32.15), was significantly higher when compared with the daytime work group.

**Conclusion:**

Shift work appears to have an association with the metabolic syndrome in female workers. Accordingly, we believe that the attention of government agencies and business owners is needed together with the individual practice of health behaviors to manage the metabolic syndrome for the prevention of cardiovascular disease in female shift workers.

## Introduction

The metabolic syndrome, a state of insulin resistance, is a syndrome that is accompanied by abdominal obesity, lipoidosis, impaired glucose tolerance, and high blood pressure [1]. After being named “syndrome X” by Reaven [2] the cluster of cardiovascular disease risk factors was named the metabolic syndrome and diagnosis criteria was first proposed by the World Health Organization (WHO) [3]. Later, in 2001, the United States National Cholesterol Education Program Adult Treatment Panel (NCEP-ATP) III emphasized the importance of the metabolic syndrome for managing cardiovascular problems and proposed standards for diagnosing the metabolic syndrome that required three or more of the following criteria to be met: abdominal obesity, hypertriglyceridemia, low high-density lipoprotein cholesterolemia, high-blood pressure, and impaired glucose tolerance [4]. In 2005, the International Diabetes Federation (IDF) proposed new diagnosis criteria that modified the NCEP-ATP III, noting the high relation to insulin resistance, and proposed abdominal obesity, an easy clinical measurement, as a diagnostic prerequisite [5]. In addition, in 2005, the American Heart Association/National Heart Lung and Blood Institute (AHA/NGLBI) modified the diagnostic criterion for impaired fasting glucose and the use of medication as a risk factor, based on NCEP-ATP III [6].

The metabolic syndrome increases the risk of diabetes and cardiovascular disease, as well as the death rate from these diseases [7]. In the United States, 34% of adults over 20 have the metabolic syndrome, and the rate continues to increase [8]. The results of an analysis of data gathered from the Korean National Health and Nutrition Examination Surveys (KNHANES) from 2007–2010 showed the prevalence rate of the metabolic syndrome in South Korea for adults over 30 to be 31.9% for men and 25.6% for women, with a total prevalence of 28.8% [9]. This is important, not only from a clinical perspective, but from a public health perspective as well [10].

While shift work is being adopted by companies for economic, technical, and social reasons, it means dividing workers into two or more groups so that they work in shifts at separate times. This definition excludes temporary or occasional night work, night duty done by daytime workers, and fixed-time work cycles in which workers do not change shifts. The method of shift work can be divided into shifts that include night work and shifts that do not, as well as 24-hour periods divided into two 12-hour or three 8-hour shifts that rotate every 4–5 days [11]. 20-25% of manufacturing workers in Europe and the United States perform shift work [12]. In South Korea, the results of a study by the Ministry of Employment and Labor in 2011 on companies with 10 or more employees (3,414 samples) revealed the proportion of shift workers to be 15.2% [13].

Human physiology has a circadian rhythm of about 24 hours, which is maintained by the interaction of environmental conditions and the biological clock [14]. The disruption of the circadian rhythm that results from shift work has been reported to lead not only to problems such as sleep disorders and exhaustion, but also to domestic life and social activity problems [15], as well as various health problems [16].

Shift work-related health problems include an increase in digestive system problems, drowsiness, exhaustion, and sleep disorders [17]. They also include a metabolic syndrome-related increase in body fat, as well as a rise in blood pressure, which, through an increase in ischemic heart disease and cardiovascular problems, has led to an increase in mortality rates [18]. In women, there are also reports of an association with breast cancer, menstrual irregularity, and infertility [19].

Research on the factors that influence the development and prevalence of the metabolic syndrome in Korean workers, as well as on the physical harm caused by shift work, has mainly focused on male workers in dockyards, electronics manufacturing, and steel-mills [20,21]. While there has been one study on female workers that examined the association of shift work duration and cardiovascular disease risk factors in university hospital nurses [22], there is still an insufficient number of studies on the association of shift work and the metabolic syndrome in female manufacturing workers.

Accordingly, this study was carried out to provide baseline data about the health management and prevention of metabolic syndrome-linked disease in female shift workers and to investigate the association between shift work and the metabolic syndrome in female workers in dye-related jobs.

## Materials and methods

1. Research objectives

Out of the 343 female workers from the 19 fabric processing businesses located in the Dyeing Industrial Complex near the city of Daegu, 257 participated in the medical examination (74.9% participation rate) in the Department of Occupational and Environmental Medicine at ‘Y’ University Hospital from April until December of 2012. Among those, excluding 3 individuals with a medical history of thyroid disease, 254 individuals were included as subjects in this study. This study was conducted in compliance with the international “Declaration of Helsinki”. The Institutional Review Board of our university hospital approved the study protocol.

2. Research methods

1) Data collection

In the process of conducting regular and special medical examinations on workers, we inquired about their past medical history, habits of exercise, smoking, drinking, type of work, and whether or not they did shift work through interview-based medical examinations. Height and weight were measured using automated height and weight measuring devices with the subjects lightly clothed and their shoes removed. For the waist circumference, we measured midway between the pelvic iliac crest and the lower hypochondrium in an upright posture, in accordance with the WHO's diagnostic criteria for abdominal obesity [23]. Blood pressure was measured with a mercury blood pressure monitor after the subject had rested for at least 4 minutes while sitting. Blood samples were collected from the study subjects between 8–10 a.m. after fasting for a minimum of 8 hours under the guidance of the company health administrator, and blood glucose, triglycerides, and high-density lipoprotein cholesterol were measured using an automated chemistry analyzer (Olympus AU5400). Shift workers were not examined on a day in which they worked a late night/early morning shift. Instead, they were examined on a day when they worked a morning or day shift.

2) Diagnostic criteria for the metabolic syndrome

For the diagnostic criteria for the metabolic syndrome, we used the modified ATP III definition proposed by the American Heart Association/National Heart, Lung and Blood Institute (AHA/NGLBI) in 2005 [5] based on the standards set in the 2001 NCEP-ATP III [4]. For the abdominal obesity criterion, we used Korean-specific waist circumference values (90 cm for men, 85 cm for women) proposed in 2005 by the Korean Society for the Study of Obesity [24]. Thus, the metabolic syndrome can be diagnosed by meeting 3 or more of the 5 criteria: a waist circumference of 85 cm and above; triglyceride levels of 150 mg/dL or greater, or taking medication to lower triglyceride levels; a high-density lipoprotein (HDL) cholesterol level under 50 mg/dL, or taking medication to raise HDL levels; systolic blood pressure at or above 130 mmHg, or diastolic blood pressure at or above 85 mmHg, or taking antihypertensive medication; fasting blood glucose of 100 mg/dL or higher, or taking medication to blood glucose.

3) Daytime workers and shift workers

Daytime workers were defined as those individuals who worked from 7–8 am to 5–6 pm, while the rest - those who worked 2 shifts of 8 hours, 2 shifts of 12 hours, or 3 shifts of 8 hours - were considered shift workers.

3. Data analysis

The collected data was analyzed using the statistical program SPSS version 18.0 (SPSS, Inc., Chicago, IL, USA). The research subjects were divided into a daytime work group and a shift work group, after which the general characteristics, physical measurements, and examination findings, as well the frequency of abnormalities for each metabolic syndrome component, were analyzed for both groups, using the chi-squared test, Fisher's exact test, and t test. In addition, in order to analyze the factors that influence the prevalence of the metabolic syndrome in female workers, we conducted a logistic regression analysis using the prevalence of the metabolic syndrome as a dependent variable, selecting only those factors that yielded a p-value of 0.25 or lower in a univariate analysis, and calculated the odds ratio and its 95% confidence interval. The level of statistical significance was set at a p-value of less than 0.05.

## Results

1. General characteristics of the research subjects

Among the research subjects, there were 71 individuals in the day work group (28.0%) and 183 in the shift work group (72.0%). The average age for the day work group was 36.4 ± 12.5 years (18–76). The shift work group, with an average age of 49.7 ± 8.6 years (21–70), was significantly older (p < 0.001). A majority of the day work group, 46 individuals (64.8%), were under 40 years old, whereas a majority of the shift work group, 102 individuals (55.7%), were 40–49 years old.

Subjects that currently smoked included 1 from the daytime group (1.4%) and 2 from the shiftwork group (1.1%). Among the individuals who consumed alcohol at least once a week, 36 (50.7%) were daytime workers. This was a higher rate of alcohol consumption (p = 0.040) compared to the 67 shift workers (36.6%) who drank at least once a week. Among the individuals who exercised regularly at least once a week, the daytime work group exercised at a higher rate (p = 0.011), with 61 individuals (85.9%), compared to the shift work group with 129 individuals (70.5%).

Concerning past disease history, 3 individuals from the daytime work group (4.2%) had a history of high blood pressure, while the number from shift work group was significantly higher, with 23 individuals (12.6%) (p = 0.049). One individual from the daytime work group (1.4%) and 2 from the shift work group (1.1%) had diabetes. None of the daytime workers had dyslipidemia, while 1 of the shift workers (0.5%) did (Table 1).

**Table 1:** **General characteristics of the subjects** (**No**(%))

**Characteristics**		**Daytime workers (n = 71)**		**Shift workers (n = 183)**		**p-value**
Age (years)	18-39	46	(64.8)	22	(12.0)	<0.001^*^
	40-49	17	(23.9)	102	(55.7)	
	≥50	8	(11.3)	59	(32.2)	
	Mean ± SD	36.4 ± 12.5		49.7 ± 8.6		<0.001^‡^
Smoking	Yes	1	(1.4)	2	(1.1)	1.000^†^
	No	70	(98.6)	181	(98.9)	
Alcohol drinking	Yes	36	(50.7)	67	(36.6)	0.040^*^
	No	35	(49.3)	116	(63.4)	
Exercise	Yes	61	(85.9)	129	(70.5)	0.011^*^
	No	10	(14.1)	54	(29.5)	
Hypertension	Yes	3	(4.2)	23	(12.6)	0.049^†^
	No	68	(95.8)	160	(87.4)	
DM	Yes	1	(1.4)	2	(1.1)	1.000^†^
	No	70	(98.6)	181	(98.9)	
Dyslipidemia	Yes	0	(0.0)	1	(0.5)	1.000^†^
	No	71	(100.0)	182	(99.5)	

*calculated by chi-squared test, † calculated by Fisher's exact test, ‡ calculated by t-test.

SD, standard deviation; DM, diabetes mellitus.

Among the businesses where the research subjects worked, 8 had under 50 employees, and 9 had between 50 and 99 employees, while 2 of the businesses had 100 employees or more, and shift work was organized in 2 or 3 shifts. Among the daytime work group, 20 people (28.2%) did office work, 16 people (22.5%) worked in research, 14 (19.7%) in administration, and 6 (8.5%) in sales. All of the subjects from the shift work group worked in manufacturing: 74 people (40.4%) worked in inspection, 36 people (19.7%) worked in processing and pre-processing, 25 (13.7%) as cutting, and 13 (7.1%) as winders.

2. Physical measurements and examination findings of the research subjects

The average and standard deviation of the waist circumference for the daytime work group and the shift work group was 71.5 ± 6.0 cm and 75.0 ± 5.5 cm, respectively, showing no significant difference. For body mass index, however the shift work group had a higher BMI at 23.9 ± 2.9 kg/m [2] versus the 22.4 ± 3.7 kg/m [2] for the daytime worker group (p = 0.043). The systolic blood pressure of each group was 117.3 ± 13.0 mmHg and 125.1 ± 17.1 mmHg, the shift work group having a significantly higher rate (p = 0.008). The diastolic blood pressure was 74.7 ± 7.5 mmHg and 77.5 ± 9.3 mmHg, showing no difference between the two groups. Fasting blood glucose levels were 85.5 ± 12.7 mg/dL and 98.4 ± 37.0 mg/dL, with the shift work group having significantly higher levels (p = 0.003). The total cholesterol for the daytime work group and the shift work group was 180.6 ± 30.8 mg/dL and 193.0 ± 35.2 mg/dL, low-density lipoprotein cholesterol was 103.2 ± 26.6 mg/dL and 112.2 ± 31.6 mg/dL, and high-density lipoprotein cholesterol was 60.9 ± 15.7 mg/dL and 59.1 ± 15.1 mg/dL, respectively, with no significant difference between the two groups. Triglyceride levels, however, were 87.8 ± 58.4 mg/dL and 105.5 ± 66.2 mg/dL, respectively, revealing significantly higher levels among shift workers (p = 0.025)(Table 2).

3. Frequency of abnormalities in the metabolic syndrome components

**Table 2:** Results of the physical examination and laboratory tests of the subjects

**Physical examinations and laboratory tests**	**Daytime workers (n = 71)**	**Shift workers (n = 183)**	**p-value**^**†**^
	**mean ± SD**	**mean ± SD**	
WC (cm)	71.5 ± 6.0	75.0 ± 5.5	0.471
BMI (kg/m [2])	22.4 ± 3.7	23.9 ± 2.9	0.043
SBP (mmHg)	117.3 ± 13.0	125.1 ± 17.1	0.008
DBP (mmHg)	74.7 ± 7.5	77.5 ± 9.3	0.139
FBS (mg/dL)	85.5 ± 12.7	98.4 ± 37.0	0.003
Total cholesterol (mg/dL)	180.6 ± 30.8	193.0 ± 35.2	0.225
LDL cholesterol (mg/dL)	103.2 ± 26.6	112.2 ± 31.6	0.117
TG (mg/dL)	87.8 ± 58.4	105.5 ± 66.2	0.025
HDL cholesterol (mg/dL)	60.9 ± 15.7	59.1 ± 15.1	0.540

WC, waist circumference; BMI, body mass index; SBP, systolic blood pressure; DBP, diastolic blood pressure; FBS, fasting blood sugar; LDL, low density lipoprotein; TG, triglyceride; HDL, high density lipoprotein.

† calculated by t-test.

Among each of the components of the diagnostic criteria, there was no significant difference between the 2 groups’ waist circumference, triglyceride levels, and high-density lipoprotein cholesterol, whereas in the case of systolic and diastolic blood pressure and fasting blood glucose, there was a significantly higher percentage of abnormal levels within the shift work group (p < 0.001). The prevalence of the metabolic syndrome among the daytime work group was 2.8%, while the shift work group had a significantly higher prevalence of 15.3% (p = 0.006) (Table 3).

4. Logistic regression analysis results according to general characteristics and shift work

**Table 3:** Prevalence of each criterion for the metabolic syndrome of the subjects

**Criterion**	**Daytime workers (n = 71)**		**Shift workers (n = 183)**		**p-value**
	**Number (%)**		**Number (%)**		
WC (≥85 cm)	4	(5.6)	13	(7.1)	0.674^*^
BP (≥130/85 mmHg) or being treated	20	(28.2)	92	(50.3)	0.001^†^
FBS (≥100 mg/dL) or being treated	3	(4.2)	45	(24.6)	<0.001^†^
TG (≥150 mg/dL) or being treated	7	(9.9)	36	(19.7)	0.061^†^
HDL (<50 mg/dL) or being treated	18	(25.4)	49	(26.8)	0.817^†^
Metabolic syndrome^‡^	2	(2.8)	28	(15.3)	0.006^†^

WC, waist circumference; FBS, fasting blood sugar; TG, triglyceride; HDL, high density lipoprotein.

*calculated by Fisher’s exact test, † calculated by × ^2^ test.

‡ Metabolic syndrome is any 3 of these 5 criteria: WC ≥ 85 cm, BP ≥ 130/85 mmHg, FBS ≥ 100 mg/dL, TG ≥ 150 mg/dL and HDL < 50 mg/dL or being medicated for the same conditions.

In the univariate analysis, the greater the age, the greater the alcohol consumption, the less a worker exercised, the greater the likelihood of a worker having the metabolic syndrome. Those who did shift work were also more likely to have the metabolic syndrome (p < 0.05). Therefore, we used age, alcohol consumption, exercise habits, and whether or not subjects did shift work, factors which had yielded p-values of 0.25 or lower in univariate analysis, as variables in a logistic regression analysis and calculated the odds ratio and its 95% confidence interval (CI) for the metabolic syndrome. In the logistic regression analysis results, in comparison with the daytime work group, the shift work group had an odds ratio of 6.30 (95% CI 1.24-32.15), while the other variables were not significant (Table 4).

**Table 4:** **Logistic regression analysis for the metabolic syndrome of female workers by general characteristics and shift work** (**N** = **254**)

**Variables**		**OR**	95% CI^*^
Age	≤39	1.00	
	40-49	0.38	0.10 - 1.46
	≥50	1.16	0.30 - 4.57
Alcohol drinking	No	1.00	
	Yes	0.59	0.20 - 1.68
Exercise	No	1.00	
	Yes	0.47	0.21 - 1.07
Shift work	No	1.00	
	Yes	6.30	1.24 - 32.15

*OR*, odds ratio.

*95% confidence interval.

## Discussion

The clinical importance of the metabolic syndrome has been revealed through many prospective studies. Ford et al. [25] analyzed prospective studies published from 1998 to 2004 and reported that the estimated relative risk of cardiovascular disease for the metabolic syndrome, as defined by NCEP-ATP III, was 1.65 (95% CI 1.38-1.99), and the estimated relative risk for diabetes was 2.99 (95% CI 1.96-4.57). This showed that the metabolic syndrome was an important risk factor for developing cardiovascular disease and diabetes. Lakka et al. [26] reported their results from monitoring middle-aged men with the metabolic syndrome for 11.4 years, finding that the relative risk of coronary artery disease was 2.9 (95% CI 1.2-7.2), while the relative risk of mortality from coronary artery disease, cardiovascular disease, and other causes was 3.6 (95% CI 1.7-7.9), 3.2 (95% CI 1.7-5.8), and 2.3 (95% CI 1.5-3.4), respectively. Boden-Albala et al. [27] conducted a follow-up study on 3,298 people for an average of 6.4 years, looking at the association of the metabolic syndrome and cerebrovascular disease. They reported an association of the metabolic syndrome with the risk of cerebral infarction, the odds in men being 1.1 (95% CI 0.6-1.9), while women had greater odds of 2.0 (95% CI 1.3-3.1). Thus, the prevention and treatment of the metabolic syndrome before the development of cardiovascular disease and diabetes is very important. Also, the metabolic syndrome increases the risk of left ventricular hypertrophy unrelated to high blood pressure, it is associated with subclinical cardiovascular disease, arteriopathy, renal disease, and atrial fibrillation [28], and it is also associated with health problems such as fatty liver disease, polycystic ovary syndrome, cholesterol gallstone, asthma, sleep disorder, and malignant tumors [29].

The prevalence rate of the metabolic syndrome in South Korean adults increases with age [30]. In this study, we looked at the prevalence of the metabolic syndrome as well as abnormal values of each component criterion for different age groups. For the age brackets of under 40, 40–49 years old, and 50 and above, blood pressure was 26.5%, 44.5%, and 61.2%, respectively; fasting blood glucose was 7.4%, 16.0%, and 35.8%; waist circumference was 7.4%, 5.0%, and 9.0%; triglyceride levels were 10.3%, 13.4%, and 29.9%; low high-density lipoprotein cholesterolemia was 22.1%, 21.0%, and 40.3%; and the prevalence of the metabolic syndrome was 7.4%, 7.6%, and 23.9%, respectively. These results showed signs of an increase in the prevalence of the metabolic syndrome and abnormalities of each metabolic syndrome component as the age brackets increased. These results were similar to those of a study on the factors related to the metabolic syndrome in dockyard and electronics manufacturing workers [21,23]. The subjects in our study aged 30 or over comprised 223 individuals (87.8%), of which 13.5% had the metabolic syndrome. This was lower than the 25.6% metabolic syndrome prevalence in women aged 30 and over reported in the Korean National Health and Nutrition Examination Surveys of 2007–2010. One reason for this discrepancy might be found in the KNHANES, which reported that odds ratio of the metabolic syndrome for full-time homemakers was 1.85 times greater than for non-homemakers. According to research by Waggoner et al. [31], the lower prevalence of the metabolic syndrome for women with jobs can be attributed to the healthy worker effect.

Smoking is an example of another factor related to the development of the metabolic syndrome. Smoking, a risk factor in coronary artery disease, increases total cholesterol and triglyceride levels, induces insulin resistance, hyperinsulinemia, and low high-density lipoprotein cholesterol, and appears to raise the risk of the metabolic syndrome [32]. In addition, smoking shows a strong correlation with the five diagnostic criteria for the metabolic syndrome proposed by ATP III. David et al. [33] reported that smoking itself is associated with a high-fat and low-fiber diet, which increases total cholesterol and triglyceride levels. As only three out of the 254 subjects in this study were smokers, the small number of subjects made the confirmation of an association between smoking and the metabolic syndrome difficult.

Through a number of studies, it has become clear that a moderate amount of alcohol consumption lowers the risk of heart disease. A moderate amount of alcohol consumption inhibits blood coagulation and platelet aggregation and also increases high-density lipoprotein cholesterol, by which it is believed to have cardioprotective effects. On the other hand, the overconsumption of alcohol is known to increase triglyceride levels and raise blood pressure [34]. Meanwhile, there are reports that, compared with men, women are more susceptible to health hazards from alcohol consumption [35]. In the United States Recommended Dietary Allowance proposed in 2005, the recommended alcohol consumption is no more than two glasses per day for men, and half of that amount for women, no more than 1 glass a day [36]. In this study as well, while the prevalence of the metabolic syndrome was higher in those who drank alcohol than in those who did not, it was not statistically significant.

Exercise is known to reduce overall metabolic risk factors, improving lipid metabolism and lowering blood pressure in patients with high blood pressure, reducing insulin resistance, and improving hyperglycemia [37,38]. In this study, while those who exercised regularly at least once a week for 30 min or more tended to have lower triglyceride and fasting blood glucose levels, higher levels of high-density lipoprotein cholesterol, and a lower prevalence of the metabolic syndrome compared with those who did not exercise, the differences were not statistically significant.

In this study, shift work was confirmed to be a risk factor for the metabolic syndrome in female workers. This relationship was also found in a study by Li et al. [39], which found that the odds ratio for the metabolic syndrome in both male and female shift workers was 1.87 (95% CI 1.13-3.08), and in the three-year follow-up study by Kawada et al. [40], the odds ratio for male double-shift workers was 1.43 (95% CI 1.05-1.95). However, a study by Puttonen et al. [41] found the odds ratio of the metabolic syndrome in men to be 1.83 (95% CI 1.13-2.96), with no meaningful difference from the prevalence in women. These findings differ from the results of our study. That shift work has a direct association with the development of cardiovascular disease can be inferred from the following studies: Research by Esquirol et al. [42,43] found through a meta-analysis of 17 studies on shift work-related health hazards that shift workers, both male and female, had 1.4 times greater odds of developing cardiovascular disease than did daytime workers. Vyas et al. [44], through a systematic review and meta-analysis of 34 experiments conducted at Canada's Western University comprising 2,011,935 subjects, found the odds ratios for myocardial infarction, cerebral infarction, and coronary artery disease in shift workers compared to daytime workers were 1.23 (95% CI 1.15-1.32), 1.05 (95% CI 1.01-1.09), and 1.24 (95% CI 1.10-1.39), respectively.

Factors such as stress, lack of sleep, and changes in health behavior are involved in the mechanism of the metabolic syndrome [45]. The association of the metabolic syndrome and stress has been confirmed by many studies [46,47]. When stress is continuously undergone, an increase in adrenocortical hormones caused by hypothalamus-pituitary-adrenal axis dysregulation leads to metabolic dysfunctions such as high blood pressure, hardening of the arteries, insulin resistance, and dyslipidemia. It also produces an anti-insulin effect, causing a rise in blood glucose levels, and can cause visceral obesity by accumulating abdominal fat [48,49]. In addition, studies have found that partial sleep restriction causes a change in the hormones that regulate the ingestion of food, ghrelin (increases after sleep restriction) and leptin (decreases after sleep restriction), which increases appetite [50], while a lack of sleep reduces glucose tolerance and insulin-sensitivity, and increases blood pressure [45]. According to research by Lee et al. [51], the stress received by shift workers is significantly high compared with that of daytime workers, the same of which is true for sleep disorders [52]. Ordinarily in humans, during the day, glycometabolism is accelerated and fat storage occurs. While sleeping at night, glucose-use lowers and lipometabolism occurs. Therefore, having to ingest food at night due to shift work, when insulin-sensitivity is at its lowest, can lead to metabolic dysfunction [45].

For reasons such as these, stress management and the management of appropriate eating habits and lifestyle habits by shift workers appears necessary in order to reduce the prevalence of the metabolic syndrome. Likewise, employers must work to prevent illness by taking actions such as giving shift workers sufficient rest time, checking the state of their health on a frequent basis, managing the working environment, and providing anti-smoking/drinking education, daily physical activities, and healthy meals. On a national level, there appears to be a need for oversight to ensure compliance with working hour requirements, night work restrictions, and vacation provision, as well as the establishment of policies to reduce the number of night-shift workers.

Among the limitations of this study were the difficulty in determining causal relationships as a cross-sectional study, and the exclusion of the investigation of the influence of socioeconomic factors on the metabolic syndrome. For example, at the time of selecting the research subjects, in order to minimize employment-related biases, we selected subjects who were working in the fabric industry. However, when we divided the subjects by office work and manufacturing work, the manufacturing jobs were mainly categorized as shift work, and office work became mostly day work. Because of this, the effect of shift work became conflated with the type of work, and our being unable to separate this was a limitation. An additional drawback was that we could not take the qualitative aspects of each variable, such as the shift work hours or the intensity of the work, into consideration.

## Conclusion

Our confirmation that shift labor can influence the prevalence of the metabolic syndrome in female workers doing such work is of value. What is needed from this point on are large-scale prospective studies on the management and prevention of the metabolic syndrome that involve more female worker subjects and that take into account socioeconomic factors as well as the other factors investigated in our study.

## Competing interests

The authors declare that they have no competing interests.

## Authors’ contributions

HHY: Study concept and design, Drafting of the manuscript. JUJ: Analysis of data. JS: Technical support. JMJ: Critical revision of the manuscript. All authors read and approve the final manuscript.
